# Supplementary material for: Content-rich biological network constructed by mining PubMed abstracts
Source: BMC Bioinformatics. 2004 Oct 8;5:147. doi: 10.1186/1471-2105-5-147 (PMC528731; doi:10.1186/1471-2105-5-147)
Supplement: Additional File 5 — The original Chilibot query results of the term "long-term potentiation (LTP)" and 22 other terms, limiting the latest references analyzed to the years 1990, 1995, 2000, and 2004. [file 1471-2105-5-147-S5.bz2 › chilibotAdditionalFile5/ltp2000/html/left.html]

 


### Chilibot Session: ltp2000

|  |  |  |  |
| --- | --- | --- | --- |
| Home | New Session | Folders | Log Out |

Image created with  aiSee 2.1  © 2003 AbsInt

View legend |
View input file |
Query history |
Image source file

|
|  |
|


---


 **a sub-network graph containing only relationships** 

above sentence level
are interactive
with weight > 1
with weight > 2
with weight > 3
with weight > 4
with weight > 5


---

**Statistics:**  

```
Searches performed: 253  
Relevant PubMed records: 10732  
PubMed records processed: 2299 (21.42%)  
Number of links found: 192   

Start  time: Sun Feb 15 21:37:59 2004   
Finish time: Sun Feb 15 21:54:48 2004
```

---

**Solitary terms:** None

---
